# Mycobacteria isolated from temperate stony corals

**DOI:** 10.17912/micropub.biology.001863

**Published:** 2026-01-22

**Authors:** Dana Ulanova, Takuma Mezaki, Satoshi Kubota

**Affiliations:** 1 Faculty of Agriculture and Marine Science, Kōchi University, Kochi, Kochi, Japan; 2 Kuroshio Biological Research Foundation, Ōtsuki Chō, Kochi, Japan; 3 Kuroshio Science Unit, Multidisciplinary Science Cluster, Kōchi University, Kochi, Kochi, Japan

## Abstract

In marine environment, actinobacteria are widely distributed in water and sediments, and form symbiotic relationships with higher organisms. In this study, we isolated 49 actinobacterial strains from three temperate stony corals *, Pocillopora damicornis* , *Acropora hyacinthus* and *Acropora muricata* . More than 60% of obtained actinobacterial isolates belonged to mycolic acid-containing genera, particularly members of the family *Mycobacteriaceae* . Our results combined with the previous studies demonstrated that these actinobacteria are frequently associated with coral hosts worldwide.

**Figure 1. Coral samples, number of isolates and phylogenetic tree of Mycobacteriaceae isolates:**
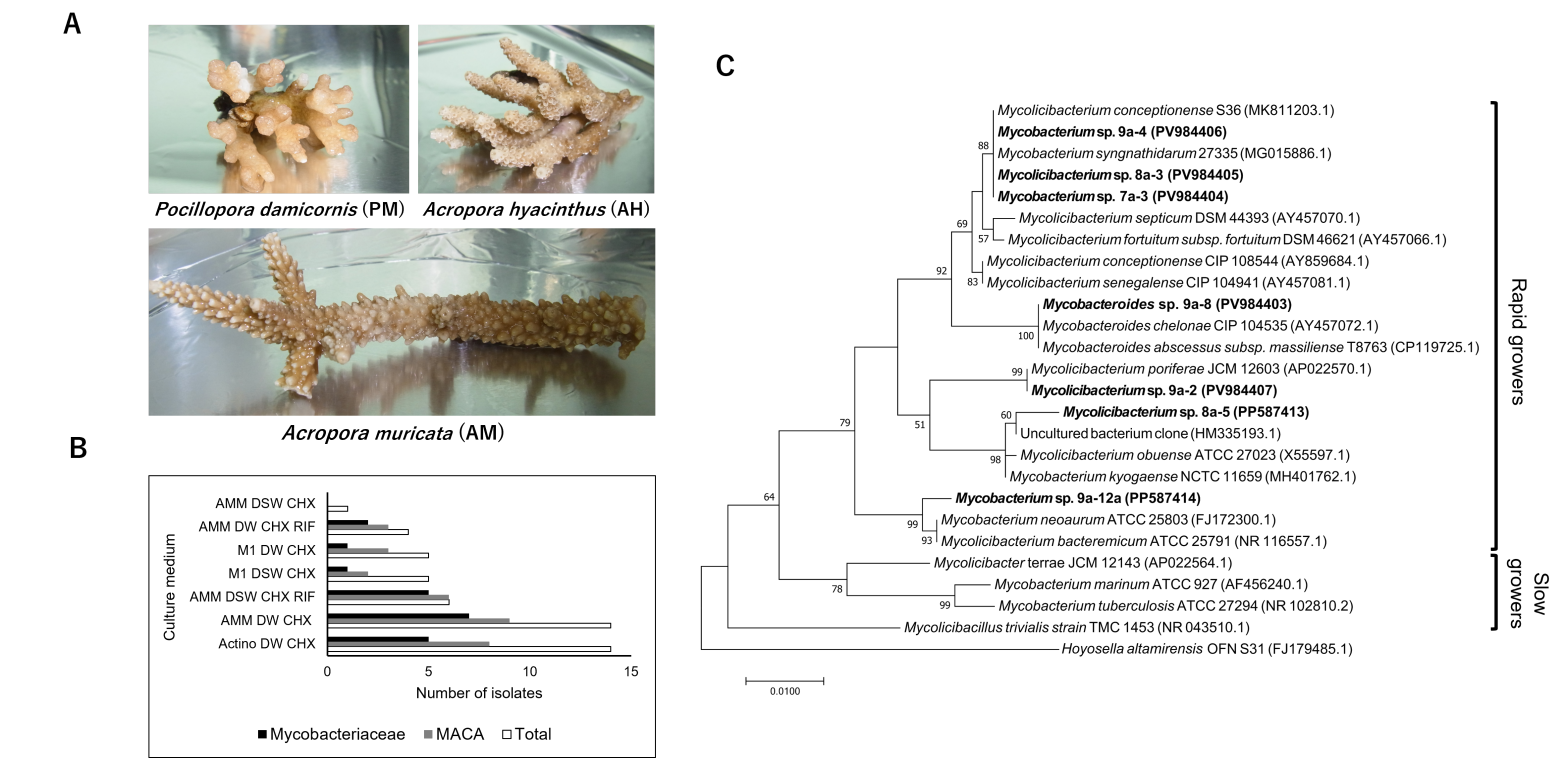
(A) Coral samples used in this study. (B) Number of total, mycolic acid-containing actinobacteria (MACA) and *Mycobacteriaceae* isolates from each type of culture medium. Media abbreviations are as given in the Methods section. (C) Maximum likelihood phylogenetic tree using 16S rRNA gene sequences (732 nucleotide positions) from *Mycobacteriaceae* isolates (in bold), their closest BLAST hits and representative strains of each clade. Species names are followed by the strain identifier and GenBank accession number. *Hoyosella altamirensis* OFN S31 was used as an outgroup. Bootstrap values >50% are shown for 1,000 replicates at the respective nodes. Rapid-growing genera– *Mycobacteroides* (“ *Abscessus-Chelonae* ” Clade), *Mycolicibacterium* (“ *Fortuitum-Vaccae* ” Clade). Slow-growing genera – *Mycolicibacter* (“ *Terrae”* Clade), *Mycolicibacillus* (“ *Triviale* ” Clade), *Mycobacterium* (“ *Tuberculosis-Simiae* ” Clade) (Gupta *et al.* 2018).

## Description

Coral reefs are critical for the survival and functioning of marine organisms and ecosystems. Stony corals, the central component of reef ecosystem, are cnidarian organisms which have a close symbiotic relationship with diverse microorganisms, forming so-called coral holobiont. Coral-associated bacteria (CAB) were detected and isolated from all parts of the coral body (mucus, tissue and skeleton) and are known to provide coral hosts with nutrients, support coral larvae settlement and help to overcome stress conditions (Sweet et al., 2021; Voolstra et al., 2024). Most of the reported CAB belongs to *Pseudomonadota, Bacillota, Actinomycetota* and *Bacteroidota* phyla (Sweet et al., 2021). While the abundance and function of several CAB, especially those from the phylum *Pseudomonadota* , have been described *(* Rosado et al., 2018; Voolstra et al *.,* 2024 *)* , the roles of many others remain unclear.

Actinobacteria are Gram-positive bacteria well-known for producing bioactive compounds, which are widely used as antimicrobial, anthelmintic and anticancer drugs in human and animal medicine. These bacteria are found in both aquatic and terrestrial environments and many of them are associated with plant and animal hosts (van der Meij et al., 2017). Actinobacteria have been detected in soft and stony corals, mainly from tropical regions, and demonstrated to be a part of the core microbiome of several coral hosts (Hernandez-Agreda et al., 2018). Since actinobacteria are potent producers of bioactive compounds, they were proposed to have a role in protecting corals against pathogens (Kuang et al., 2015). Yet, the diversity, cultivability, and functional roles of coral-associated actinobacteria, particularly in non-tropical environments, are poorly understood. In this study, we aimed to address the knowledge gap regarding actinobacteria associated with corals from temperate regions. To achieve this, we isolated and identified actinobacteria from three stony coral species commonly found in the temperate marine environment of Kochi Prefecture, Japan: *Pocillopora damicornis* (PD), *Acropora hyacinthus* (AH) and *Acropora muricata* (AM).

In total, 49 actinobacteria-like colonies were isolated from fragments of three stony coral species ( Fig. 1A ). The highest number of colonies were isolated from AMM and Actinomycete isolation agars prepared with addition of distilled water and cycloheximide (CHX), and the lowest from AMM agar prepared using seawater and CHX ( Fig. 1B ). 16S rRNA gene sequence analysis classified bacterial isolates into 14 actinobacterial genera. The highest number of actinobacterial colonies (39) and genera (11) were obtained from *A. muricata* (Table 1). Interestingly, most of the isolated colonies (31) belonged to mycolic acid-containing actinobacteria (MACA): *Gordonia* (5), *Rhodococcus* (5), and representatives of the *Mycobacteriaceae* family (21).

Strains of the *Mycobacteriaceae* family, *Mycobacterium* , *Mycolicibacterium* or *Mycobacteroides* spp. (Gupta et al., 2018), were isolated from each coral host (Table 1). Based on 16S rRNA gene analysis, our isolates were closely related to the rapidly growing (forming visible colonies in less than 7 days) genera *Mycolicibacterium* spp. and *Mycobacteroides* spp., which are saprophytes widely distributed in nature (Brown-Elliot and Philley, 2017, Gupta et al., 2018) ( Fig. 1C ). The isolates related to *Mycolicibacterium* spp. strains were obtained from all coral hosts, while the later was isolated only from *A. muricata* . Interestingly, no slow-growing mycobacterial species (requering more than 7 days to form visible colonies (Gupta et al. 2018)) were detected in this study.

In this study, we used three media supplemented with distilled water or seawater, and two sets of antibiotics. Many marine bacteria, incl. actinobacteria, prefer seawater-based media for growth under the laboratory conditions. However, we obtained the highest number of actinobacterial isolates from distilled water-based media. One reason for this can be the overgrowth of seawater-based media by fast-growing marine bacteria, which form colonies within 1-2 days at 28°C. These fast growers outcompete actinobacterial colonies, which generally require more than two days to form. Rifampicin helps to suppress fast growers but inhibits some actinobacterial strains (Freel et al., 2012). Our results indicate that use of distilled water-based media may increase the isolation success rate of actinobacteria from marine environments.

Most of isolates from this study contain mycolic acids (MAs) - long fatty acids present in cell envelops, which provide protection to bacterial cells from environmental stresses (Dover et al., 2021). In mycobacteria, MAs were demonstrated to play a role in host immune system stimulation and biofilm formation (Marrakchi et al., 2014). *Rhodococcus* sp. and *Gordonia* sp., are known for their biodegradation and bioremediation properties (Van Der Geize and Dijkhuizen, 2004; Drzyzga, 2012). Such degradation capabilities may be used in coral-microbe symbiosis for the host protection from the pollutants present in the environment (Mahmoud and Kalendar, 2016). In addition, MACA often produce colored pigments, carotenoids, which protect cells from environmental stresses (Saubenova et al., 2024). Such pigmented bacteria may also provide protection to coral holobionts (Galasso et al., 2017).

Here, we newly report a number of rapidly growing *Mycobacteriaceae* strains cultured from three species of temperate stony corals. Mycobacteria were isolated from several tropical coral species (Li et al., 2014, 2022; Siro et al., 2022) and found to present one of core microbiome members in some corals (Hernandez-Agreda et al., 2018). Similarly to our study, Li et al. (2014) isolated fast-growing mycobacteria from *Porites lutea* samples collected at Luhuitou fringing reef. A report on oil-degrading bacteria from corals inhabiting oil-polluted areas of the Arabian Gulf, described the *Mycobacterium* sp. strain with crude oil-degrading ability from mucus sample of *Acropora clathrata* (Al-Dahash and Mahmoud, 2013). The authors suggested that this bacterium may participate in the protection of coral from oil pollution together with other oil-degrading bacteria. However, details on the phylogeny and function of the *Mycobacterium* genus in coral health and/or disease remain to be understood.

In conclusion, this study provides new insights into the diversity and distribution of coral-associated mycobacteria from temperate marine environments. Our results indicate that these actinobacteria may have a global distribution across different climate zones. This study is, however, limited to a few samples and more culture-dependent and independent research needs to be done to clarify the role of mycobacteria in coral hosts.

Table 1. Representative bacterial isolates from each coral host obtained in this study and their closest BLAST hits

**Table:** 

Coral host	Isolate name&nbsp;	Accession number	Isolation medium	The closest BLAST hit&nbsp;	e-value	Query coverage	Percent identity&nbsp;&nbsp;	Accession number&nbsp;of the BLAST hit
*Pocillopora damicornis*	7a-1&nbsp;	&nbsp;PV984411	Actino DW CHX	*Streptomyces microflavus* &nbsp;	0	100	100	CP054926.1&nbsp;
	7a-2&nbsp;	PV984402	Actino DW CHX	*Micrococcus endophyticus* &nbsp;	0	100	100	MK578827.1&nbsp;
	7a-3&nbsp;	PV984404	Actino DW CHX	*Mycobacterium syngnathidarum* &nbsp;	0	100	100	MG015886.1&nbsp;
	7a-4b&nbsp;	PV984401	M1 DW CHX	*Micromonospora* sp. &nbsp;	0	100	100	GQ339909.1&nbsp;
	7b-1a&nbsp;	PV984408	Actino DW CHX	*Pseudonocardia* sp.&nbsp;	0	100	100	MN631183.1&nbsp;
*Acropora hyacinthus*	8a-3&nbsp;	PV984405	AMM DSW CHX RIF	*Mycolicibacterium conceptionense* &nbsp;	0	100	100	MK811203.1&nbsp;
	8a-5&nbsp;	PP587413	AMM DW CHX	Uncultured bacterium&nbsp;	0	99	99.52	HM335193.1
				*Mycobacterium kyogaense*	0	100	99.41	MH401762.1
*Acropora muricata*	9a-2&nbsp;	PV984407	AMM DW CHX RIF	*Mycolicibacterium poriferae* &nbsp;	0	100	100	AP022570.1&nbsp;
	9a-3&nbsp;	PV984399	AMM DW CHX RIF	*Gordonia* sp. &nbsp;	0	100	100	DQ448776.1&nbsp;
	9a-4&nbsp;	PV984406	AMM DW CHX RIF	*Mycobacterium syngnathidarum* &nbsp;	0	100	100	MG015886.1&nbsp;
	9a-5a&nbsp;	PV984409	Actino DW CHX	*Pseudonocardia* sp. &nbsp;	0	99	100	EU928984.1&nbsp;
	9a-7&nbsp;	PV984410	Actino DW CHX	*Rhodococcus kroppenstedtii* &nbsp;	0	100	100	MK443065.1&nbsp;
	9a-8&nbsp;	PV984403	Actino DW CHX	*Mycobacteroides abscessus* subsp. *massiliense* &nbsp;	0	100	100	CP119725.1&nbsp;
	9a-10b&nbsp;	PP587412	AMM DW CHX&nbsp;	*Microbacterium* sp.&nbsp;	0	100	99.88	MN620398.1&nbsp;
	9a-12a&nbsp;	PP587414	AMM DW CHX&nbsp;	*Mycolicibacterium bacteremicum* &nbsp;	0	100	99.38	NR_116557.1&nbsp;
	9a-24&nbsp;	PV984400	M1 DSW CHX	*Nocardioides marmoraquaticus* &nbsp;	0	100	100	JN615437.2&nbsp;
	9a-26&nbsp;	PP587411	M1 DSW CHX	*Agrococcus* sp. &nbsp;	0	100	99.52	CP027942.1&nbsp;
	9a-30&nbsp;	PV984398	AMM DSW CHX&nbsp;	*Brachybacterium* sp.&nbsp;	0	100	100	MN704068.1&nbsp;
	9b-4a&nbsp;	&nbsp;PP587415	AMM DW CHX&nbsp;	*Serinicoccus marinus* &nbsp;	0	100	99.51	CP043808.1&nbsp;

## Methods

Sample collection and bacterial isolation

Coral samples were collected on October 2019 by SCUBA diving offshore Nishidomari, Otsuki town, Kochi (32°46'41.0"N 132°43'54.9"E, water depth 3 m, temperature 25°C). The collected fragments were stored in local seawater at ambient temperature and processed on the next day of collection. Coral fragments were washed twice in sterile seawater to remove loosely attached bacteria and crushed in an alcohol-sterilized mortar. The obtained slurry was directly plated on following agar media: 1) AMM (Jensen et al., 2005) prepared using distilled water (DW) or deep seawater (DSW), supplemented with CHX (100 mg L ^-1^ ) to suppress fungal growth; 2) AMM prepared using DW or DSW, supplemented with CHX (100 mg L ^-1^ ) and rifampicin (RIF, 5 mg L ^-1^ ) to suppress fungal and fast-growing bacteria growth; 3) M1 (10x diluted AMM) prepared using DW or DSW, supplemented with CHX (100 mg L ^-1^ ); and 4) DIFCO ^TM^ Actinomycete isolation agar (BD) (Actino) prepared using DW and supplemented with CHX (100 mg L ^-1^ ). Inoculated plates were cultivated at 28°C for up to one month. Actinomycete-like colonies were transferred to fresh culture plates of the same medium until pure cultures were obtained. Colonies were considered to be actinobacteria-like if spores and/or aerial mycelium were formed (sporulating actinobacteria (e.g., *Streptomyces* )) or colonies had dry or waxy appearance with pink, light or dark-yellow coloration (e.g., *Mycobacterium* , *Rhodococcus* ).

Strain identification

The 16S rRNA gene amplification reaction mixture contained 1× EmeraldAmp Max PCR master mix (TaKaRa) and 0.3 μM of 16Seu27F (Čermák et al., 2008) and 1492R (Lane D.J., 1991) primers. A bacterial colony was added to the mixture by a toothpick. The cycling condition used was initial denaturation at 95 °C for 2 min, followed by 30 cycles of 94 °C for 30 s, 55 °C for 30 s and 72 °C for 90 s. DNA sequencing was performed by ABI PRISM™ 3100 Genetic Analyser, according to manufacturer’s recommendations. Sequences were processed using Geneious sequence analysis software ver. 5.5.4 (Kearse et al., 2012) and analyzed using the BLAST program (Altschul et al *.* , 1990) (blastn, nucleotide collection database, as on April 1 ^st^ , 2024). The 97% percent identity and 90% query coverage were used as cutoffs when evaluating the blastn hits. Phylogenetic trees were constructed using MEGA7 software (Kumar et al., 2016).

16S rRNA gene sequences of the representative isolates were deposited in the GenBank under accession numbers PP587411- PP587415 and PV984398-PV984411.
